# Amyloid β-Induced Redistribution of Transcriptional Factor EB and Lysosomal Dysfunction in Primary Microglial Cells

**DOI:** 10.3389/fnagi.2017.00228

**Published:** 2017-07-19

**Authors:** Xingzhi Guo, Peng Tang, Li Chen, Peng Liu, Chen Hou, Xin Zhang, Yue Liu, Li Chong, Xiaoqing Li, Rui Li

**Affiliations:** Department of Neurology, Shaanxi Provincial People's Hospital, and the Third Affiliated Hospital, Xi'an Jiaotong University School of Medicine Xi'an, China

**Keywords:** microglia, amyloid, degradation, TFEB, lysosome

## Abstract

Impaired clearance of Amyloid β (Aβ) by microglia in the brain may be associated with the senile plaque formation, a pathological hallmark relevant to Alzheimer's disease. Microglial cells in the brain are not able to efficiently degrade Aβ, suggesting that microglial lysosome impairment may occur. However, the mechanism of Aβ-induced impairment of microglia remains poorly understood. We observed the effects of Aβ on the trafficking of nuclear transcriptional factor EB (TFEB), a master regulator of lysosome biogenesis, and the expression of a downstream osteoporosis-associated transmembrane protein 1 (OSTM1), a vital molecule involved in lysosome acidification in primary microglial cells. Aβ_1−42_ but not Aβ_42−1_ resulted in a significant release of tumor necrosis factor-α in primary microglia, but the total cellular TFEB was not changed. Further, Aβ induced a dose-dependent reduction of the TFEB in the nucleus of primary microglial cells, coincident with the increase in the plasma, as revealed by Western blot and confocal microscopy. In addition, a dramatic decrease of OSTM1 expression was observed in the Aβ-challenged microglial cells, along with the intracellular pH steady state, indicating the inadequate lysosomal acidification. These data suggest that Aβ might result in a lysosomal dysfunction via inhibiting nuclear TFEB translocation in microglial cells.

## Introduction

Alzheimer's disease (AD) is a neurodegenerative illness accounting for approximately 50–70% of late-onset dementia (Rademakers and Rovelet-Lecrux, 2009). A universal hallmark of Alzheimer's disease is the presence of extracellular senile plaque consisting of aggregated fibrillar β-amyloid (fAβ) (Ittner and Götz, 2011). Studies have demonstrated that cerebral defects in the clearance of the misfolded Aβ contributed to the Aβ accumulation (Majumdar et al., 2007; Hickman et al., 2008; Mawuenyega et al., 2010). Lysosomes are the key digestive organelle of the cell and can degrade a wide variety of structurally diverse substances including misfolded Aβ. Moreover, microglial cell, as the native immune cell in the CNS, is crucially involved in phagocytosis and degradation of misfolded Aβ (Lee and Landreth, 2010; Fu et al., 2012). However, most studies found that microglial cells were incapable of digesting engulfed fAβ, since the lysosomal acidification is inadequate for the digesting process, thus strongly suggesting the involvement of lysosomal dysfunction in the impaired fAβ degradation (Majumdar et al., 2007; Umeda et al., 2011). Evidences from both *in vitro* and *in vivo* showed that microglia could not effectively clear the fAβ in the brain, due to the lysosomal dysfunction (Hickman et al., 2008; Lee and Landreth, 2010; Perry et al., 2010). Furthermore, defective autophagy was a common phenomenon in AD mice, suggesting that the lysosomal dysfunction was involved in autophagy impairment (Wolfe et al., 2013). However, the mechanisms underlying the Aβ-induced lysosomal dysfunction remain unclear.

Transcription factor EB (TFEB), a transcription factor that coordinates the expression of lysosomal hydrolases, membrane proteins biogenesis involving in lysosomal function and autophagy (Sardiello et al., 2009), is a key regulator of lysosomal function (Sardiello and Ballabio, 2009). TFEB translocation from cytoplasm to nucleus has been shown to increase the number of lysosomes and promote protein clearance under various conditions of aliments involved in protein misfolding, including Huntington's disease, Parkinson's disease and Pompe's disease (Dehay et al., 2010; Tsunemi et al., 2012). Moreover, osteopetrosis-associated transmembrane protein 1 (OSTM1), in cooperation with chloride channel 7 (CLCN7), is essential for regulating lysosomal pH and Aβ clearance (Lange et al., 2006; Majumdar et al., 2011; Spampanato et al., 2013). Study in osteoclast showed that the expression of OSTM1 and CLCN7 was closely regulated by the nuclear translocation of TFEB (Lacombe et al., 2013). In this study, we observed the effects of Aβ stimulation on the activation of microglia and lysosomal function to elucidate the role of TFEB nuclear translocation and OSTM1 expression in the Aβ-induced lysosomal dysfunction, and to explore the possible mechanisms of compromised Aβ clearance in the microglial cells.

## Methods

### Materials

Aβ_1−42_, Aβ_42−1_, Hoescht-32258, dimethyl sulfoxide (DMSO), and bovine serum albumin (BSA) were obtained from Sigma (St. Louis, MO). DMEM-F12 and heat-inactivated endotoxin-free fetal bovine serum (FBS) were purchased from Hyclone (Logan, UT). Cell viability was evaluated with a Cell Counting Kit-8 (CCK8) assay (Dojindo Molecular Technologies, Inc.). Nuclear Protein Extraction Kit (78833) was from Pierce (Thermo Fisher, USA). Antibody against LAMP1 (ab25630) and Iba1 (ab5076) were from Abcam (Cambridge, MA, USA). Rabbit polyclonal antibody against TFEB (catalog no. sc-48784) and goat polyclonal antibody against OSTM1 (catalog no. sc-168858) were from Santa Cruz. Antibody against PCNA (catalog no. 2586), rabbit polyclonal antibody against GAPDH (catalog no. 5174) and all the secondary antibodies used in the experiment were purchased from Cell Signaling Technology (Beverly, MA). Rat TNF-α ELISA kit was purchased from BD Bioscience (catalog no. 560479).

### Microglial culture and viability

Purified primary microglial cells were obtained from cerebral cortices of neonatal Sprague-Dawley rats (1-day old), as previously described (Saura et al., 2003). All experimental protocols have been approved by the Institutional Animal Care and Use Committee of Xi'an Jiaotong University. After mechanical and chemical dissociation, cerebral cortical cells were suspended in DMEM-F12 medium supplemented with 10% FBS and seeded at a density of 5 × 10^6^ cells. The cells were cultured in 5% CO_2_ at the 37°C incubator, and the medium was changed every 4–5 days. After 2 weeks, when the mixed culture cells were grown to confluence, mild digestive method was used to harvest purified primary microglia (Saura et al., 2003), maintained in DMEM-F12 supplemented with 2% FBS for 24 h before further experimental treatments. The purity of microglial cells was evaluated by 1,1′-dioctadecyl-3,3,3′,3′-tetramethyl-indocarbocyanine per chlorate (DiI-Ac-LDL) labeling, a cell marker for living microglia, showing a 98% in purity. Cell viability was determined by the CCK-8 assay. Briefly, primary microglia cells were seeded in 96-well culture plates at a density of 5 × 10^4^ cells per well and cultured 24 h with 100 μl medium for stabilization. Cells were treated with different concentration of Aβ_42−1_ or Aβ_42−1_ as indicated concentration for 24 h. CCK8 (10 μl) solution was added to each well and incubated for further 2 h. The highly water-soluble tetrazolium salt (WST-8) from CCK8 kit was turn into a yellow-color formazan dye by microglial cells. Finally, the optical absorbance was measured at 450 nm with a microplate reader and high optical density indicated high cell viability. All the presented data were normalized to a control.

### Preparation of fibril-enriched Aβ

Aβ_1−42_ peptides were dissolved in sterilized and distilled water to make a stock solution at a concentration of 0.1 mM and incubated at 37°C for 7 d to allow peptide aggregation and fibril formation (Lorenzo and Yankner, 1994; Reed-Geaghan et al., 2009). Amyloid fibril-enriched preparation was obtained by Aβ peptide incubated in water for at least 7 days. Fibrillar form of the peptide was confirmed by its ability to change thioflavine T fluorescence spectra, as described elsewhere (Hudson et al., 2009). Reverse peptides Aβ_42−1_ were prepared with the same method serving as the negative control.

### TNF-α and mRNA measurement

Levels of TNF-α secreted by microglia in the supernatants, a sensitive marker for microglia activation, were determined after the challenge of Aβ_1−42_ or Aβ_42−1_ for 24 h, by using an ELISA assay kit. Procedures were performed following the manufacturer's instruction manual. TNF-α mRNA was detected by using real-time PCR.

### Western blot analysis

Microglial cells were treated with Aβ (5 and 10 μM) or 10 μM Aβ_42−1_ as control and then lysed in RIPA lysis buffer, followed by the extraction of total cellular, nuclear, or cytoplasmic proteins by using protein extracting kits. After the protein concentrations were measured by the BCA protein assay reagents, equal amounts of proteins (15 or 20 μg) were subjected to SDS-PAGE by using 12% for TFEB and LAMP1 and 15% for OSTM1 polyacrylamide gels. Electrophoresed proteins were then transferred to polyvinylidene difluoride membranes and blocked with 5% (w/v) non-fat dry milk in PBS or rabbit serum for 1.5 h at ambient temperature before the incubation overnight at 4°C with various primary antibodies. After extensive washing, the appropriate HRP-conjugated secondary antibody (1: 2,000, CST) diluted in PBS containing 0.1% Tween 20 was added for 1 h. Blots were developed with the luminal reagent (Thermo Scientific). Integrated optical density (IOD) measurement and analysis were conducted with the software Gel-Pro Analyzer. The primary antibodies used were as follows: anti-TFEB (1:500), anti-OSTM1 (1:1,000), anti-LAMP1 (1 μg/ml), anti-Iba1 (1 μg/ml), anti-GAPDH (1:5,000) and anti-PCNA (1:1,000). GAPDH was used for internal control of total protein and PCNA for control of nuclear protein.

### Confocal microscopy analysis

Purified microglia were rinsed with PBS and suspended with DMEM-F12. Cell suspension was subjected to centrifugation at 1,000 rpm for 8 min. The supernatant fluids were discarded and the cells were resuspended with pipettes in the remaining drops. Two small drops of the suspension were dripped on a cover slip in the plate to make it evenly distributed over the surface before adding DMEM-F12 containing 2% FBS into the plate 1 h later. After microglia was returned to the resting state, treatment was given as described above. The cover slips were rinsed three times and fixed for 20 min, permeabilized with 0.3% Triton X-100, blocked with donkey serum, and then incubated with polyclonal antibodies against TFEB and OSTM1 or LAMP1 for one night at 4°C. The cover slips were washed three times for over 15 min, then incubated for 1 h at room temperature in the darkroom with a FITC-conjugated donkey anti-rabbit IgG against TFEB antibody (1:100, CoWin Biotech, catalog no. cw-0219) and a Cy3-conjugated donkey anti-goat IgG against OSTM1 antibody (1:100, CoWin Biotech, catalog no. cw-0216) or FITC-conjugated donkey anti-mouse IgG against LAMP1 antibody (1:100, CoWin Biotech, catalog no. cw-0224). Control plates were incubated with secondary antibodies only. The cover slips were rinsed three times for over 20 min and then incubated for 10 min with Hoescht-32258. Images under a laser scanning confocal microscopy were collected. All the antibodies used above were diluted with donkey serum.

### Microglia intracellular lysosomal pH detection

Intracellular pH of microglia was detected using fluorescent dye-based pHrodo Red AM intracellular pH indicator (Invitrogen, Carlsbad, CA). The excitation and emission wavelength of 560 nm and 580 nm was used in a Gemini EM microtiter plate reader (Molecular Devices). Microglial cells were harvested and maintained in the 96-well plate, with DMEM-F12 containing 2% FBS at least for 2 h for the return of a resting state. After treatment with Aβ of the indicated concentration and indicated time, microglia cells were rinsed with live cell imaging solution and dyed with pHrodo, according to the manufacturer's protocol, followed by the intracellular pH measurement with a Gemini EM microtiter plate reader. Forskolin (100 μM), a protein kinase A (PKA) activator, capable of enhancing lysosomal acidification in microglial cells, was served as positive control. Microglia intracellular pH values were determined by the arbitrary pH ratios of light excited at 560 nm and emitted at 580 nm with pH calibration curve kit (Invitrogen).

### Statistical analysis

The data were analyzed by one-way analysis of variance (ANOVA) followed by *post hoc*-comparison using Bonferonni's method with SPSS 18.0. All data were normally distributed. Data were expressed as mean ± SEM shown by error bars. Differences were considered statistically significant when *P* < 0.05.

## Results

### The effects of Aβ on microglial activation and TFEB

**Figure 1:**
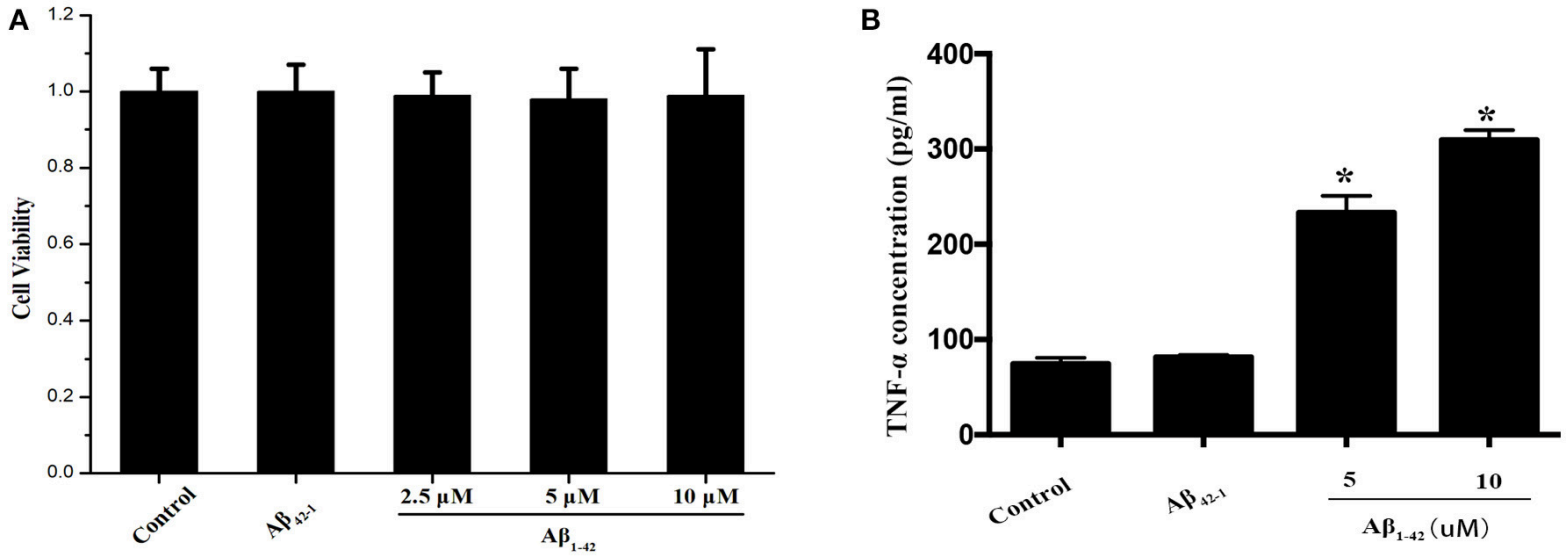
Microglia activation by Aβ. The cell viabilities of microglia among Aβ (2.5, 5, and 10 μM) and Aβ_42−1_ treated groups were similar to control group **(A)**. After incubated with Aβ for 24 h, the supernatants from primary microglia were collected to measure the TNF-α level. Comparing to normal control, administration of Aβ significantly increased the expression of TNF-α in a dose-dependent manner **(B)**. ^*^P < 0.05 (compared with resting microglia).

**Figure 2:**
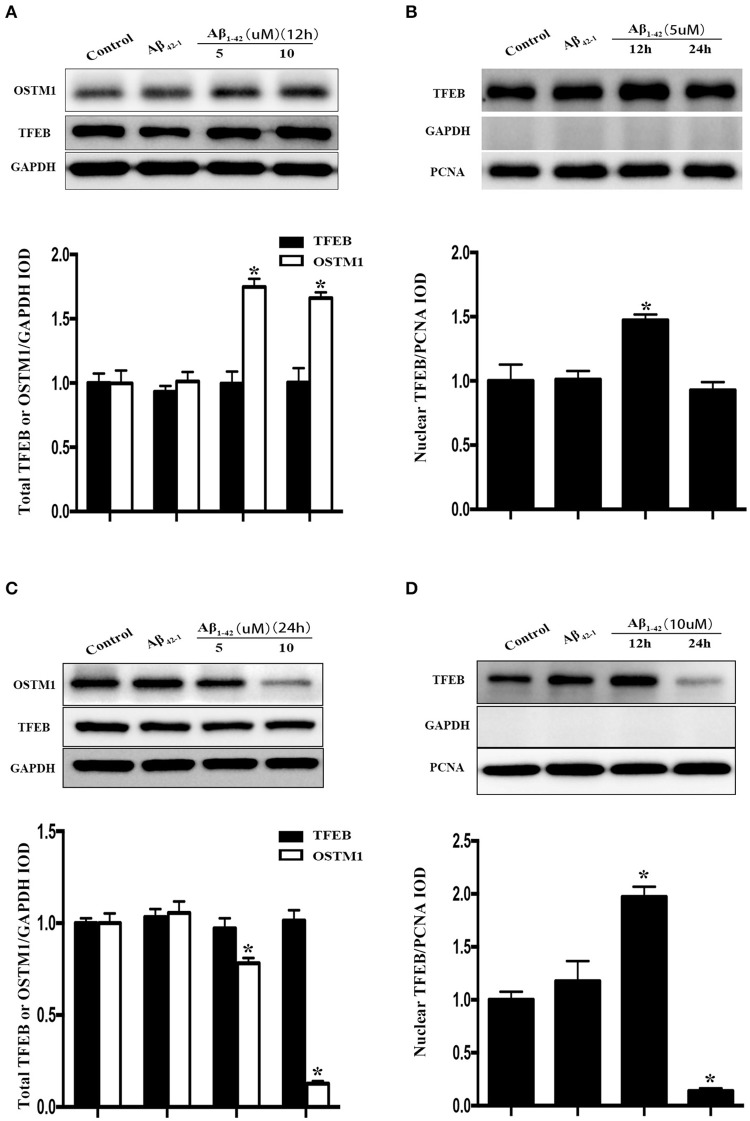
Cellular distribution of TFEB and OSTM1 in Aβ-treated microglia. After being incubated with Aβ of the indicated concentration for 24 h, primary microglial cells were lysed with RIPA buffer. Nuclear proteins were extracted to run SDS-PAGE for Western blot analysis. Treatment with Aβ (5 μM or 10 μM) and control reverse peptides Aβ_42−1_for 12 h did not alter the expression of total TFEB in microglia compared to that in resting microglia, but induce a significant increase of total OSTM1 **(A)**. Nucleus TFEB in microglia was transiently increased after treated with 5 μM Aβ for 12 h **(A)**, and then decreased to the baseline in 24 h **(B)**. Further analysis for TFEB and OSTM1 in the cytosol and nucleus revealed that the distribution of TFEB in the nucleus was reduced in microglia after treatment with Aβ for 24 h in a dose-dependent manner **(C,D)**, with a dramatical decrease of OSTM1 in the cytosol. TFEB or OSTM1 protein levels were normalized to that of GAPDH for cytoplasmic or PCNA for nuclear protein. Values were obtained from three independent experiments. ^*^P < 0.05, (compared with resting microglia).

Purified microglial cells were collected after treatment with Aβ and cell extracts were run on SDS-PAGE for immunoblotting to detect total TFEB level. The cell viability of primary microglia was not altered by Aβ_1−42_ ranging from 2.5 to 10 and 10 μM Aβ_42−1_, as indicated by CCK-8 assay (Figure 1A). After being incubated with Aβ (5 or 10 μM) for 24 h, microglial cells were activated and transformed into amoeboid morphology (Figure S1). Meantime, both 5 and 10 μM Aβ_1−42_ induced significant increase of TNF-α in the supernatant, indicating a state of microglia activation (Figure 1B). The expression of interleukin 1 (IL-1), interleukin 6 (IL-6) and TNF-α mRNA was also increased after incubated with Aβ_1−42_ (Figure S2). Importantly, no difference of total cellular TFEB in microglia was found among control and those treated with Aβ_1−42_ or Aβ_42−1_ in 12 and 24 h (Figures 2A,C).

### The effects of Aβ on the distribution of TFEB in microglia

**Figure 3:**
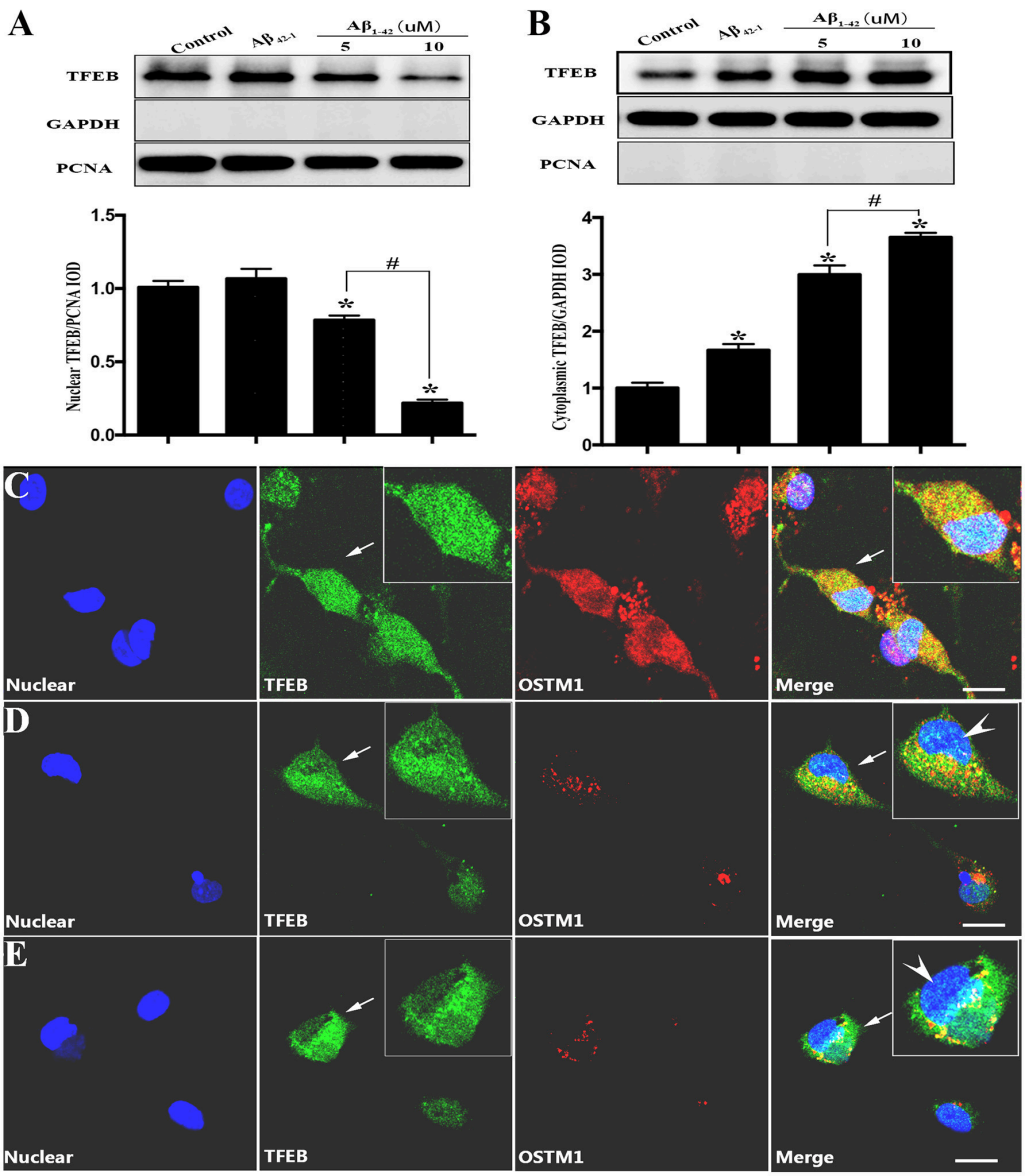
TFEB redistribution induced by Aβ. Equal nuclear proteins (15 μg) were separated on 12% SDS-PAGE, and then analyzed by immunoblotting with polyclonal antibodies against TFEB. The result **(A)** showed a significant reduction of TFEB expression in nuclear compartment after being incubated with Aβ (5 and 10 μM) by approximately 20 and 80%, respectively, as compared with control (vehicle). Conversely, the cytoplasmic proteins analysis **(B)** showed that Aβ increased the level of TFEB located in cytoplasm. Controls of nuclear and cytoplasm extraction were performed by an analysis of nuclear marker PCNA and GAPDH respectively. Furthermore, a triple-staining confocal microscopy was applied to scan microglial nucleus (blue), TFEB (green) and OSTM1 (red) after microglia was treated with Aβ for 24 h. An even distribution of TFEB in both cytoplasm and nucleus was observed in resting microglial cells **(C)**. Significant dose-dependent nuclear export of TFEB and inhibition of OSTM1 were observed in response to 5 μM **(D)** and 10 μM **(E)** Aβ. The high magnification image showed that TFEB was mainly sequestrated in cytoplasm (arrowhead) and the expression of OSTM1 decreased in primary microglia. Values were obtained from three independent experiments. ^*^P < 0.05 (compared with resting microglia). #*P* < 0.05 (compared with the indicated group). Scale bars = 25 μm.

Since TFEB translocation into nucleus is a key process in the enhancement of lysosomal function (Sardiello et al., 2009), we thus explored the influence of Aβ on nuclear translocation of TFEB. After the addition of Aβ_1−42_ or Aβ_42−1_ for 12 or 24 h in purified primary microglia, the nucleus protein and cytoplasmic protein were extracted by using the nucleus protein extracting kit. Treatment with Aβ_1−42_ (5 or 10 μM) but not Aβ_42−1_ resulted in a transient increase of TFEB within nuclear part of microglia at 12 h (Figure 2B), followed by a sustained efflux, leading to a significant increase of plasma counterpart (Figure 2D). Western blot analysis showed that TFEB were predominantly located in the nucleus of resting microglia, while the level of TFEB in the cytoplasmic compartment was relatively low (Figures 3A,B). In addition, the challenge of Aβ significantly reduced the level of nuclear TFEB in a dose dependent manner with 20% inhibition for 5 μM and 80% inhibition for 10 μM (Figure 3A). In parallel, incubation with Aβ for 24 h strongly elevated the levels of cytoplasmic TFEB in microglia by approximately 270% at 5 μM and 360% at 10 μM (Figure 3B), respectively.

### Confocal microscopy analysis for the effects of Aβ on TFEB distribution and OSTM1 expression

To further confirm the finding that Aβ-induced changes of TFEB and OSTM1, purified microglial cells were incubated with Aβ_1−42_ (5 or 10 μM) for 24 h. Antibodies against TFEB and OSTM1 were used to label the corresponding proteins. Confocal microscopy analysis revealed that Aβ addition resulted in a remarkable retention of TFEB from nuclear compartment, accompanied by a significant decrease of OSTM1 in microglia (Figures 3C–E).

### Effects of Aβ on LAMP1 expression in microglia

**Figure 4:**
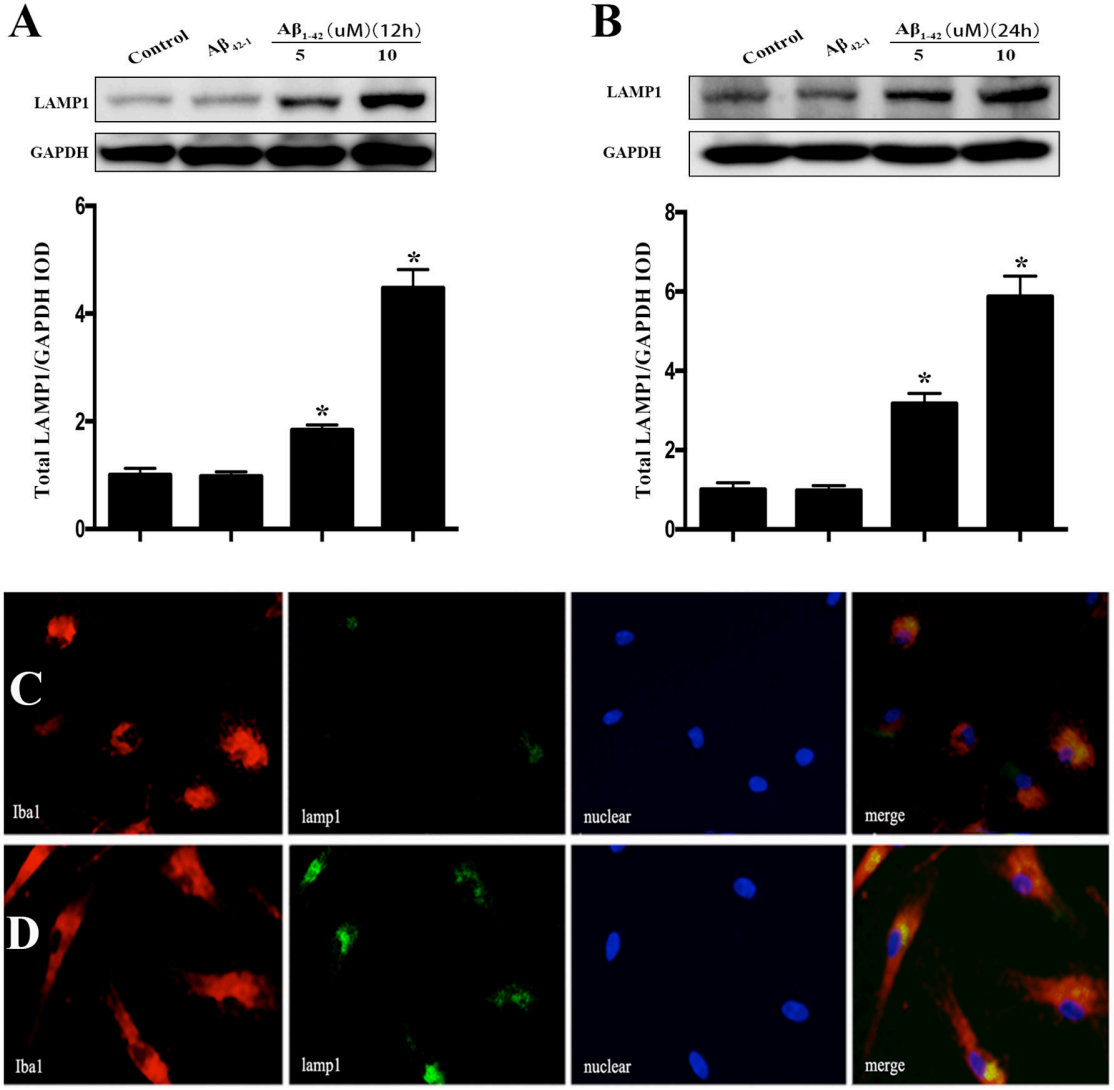
Aβ increased the expression of LAMP1 in primary microglia. After incubated with Aβ for 12 or 24 h, the primary microglial cells were lysed with RIPA buffer to extract total protein for Western blot analysis. Comparing to vehicle or control reverse peptides Aβ_42−1_, incubation microglia with Aβ (5 and 10 μM) for 12 h dramatically increase the expression of LAMP1 **(A)**. Further analysis showed that administration of Aβ for 24 h continued to up-regulate the LAMP1 levels **(B)**. As compared to control **(C)**, the cellular immunofluorescence showed that Aβ increased the expression of LAMP1 (green) in the cytoplasm of primary microglia compared to control **(D)**. The microglial cells were stained with its marker Iba1 (red). The LAMP1 protein levels were normalized to that of GAPDH. ^*^P < 0.05.

After incubated with Aβ_1−42_ (5 or 10 μM) and Aβ_42−1_ for 12 h, total protein was extracted to analyze the expression of LAMP1. The results showed that both dose of Aβ dramatically increased the LAMP1 levels (Figure 4A), which was consistent to the Western blot results of OSTM1 and nucleus TFEB. Unexpectedly, treatment of microglia with Aβ for 24 h continued to raise the level of LAMP1 (Figure 4B). In addition, the results from cellular immunofluorescence also illustrated an increased expression of LAMP1 in microglial cytoplasm at 24 h (Figures 4C,D).

### Inadequate lysosomal acidification induced by Aβ

**Figure 5:**
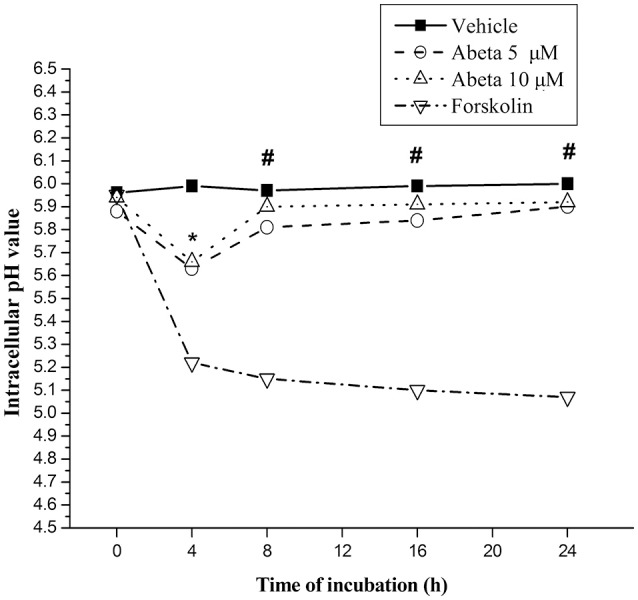
Effects of Aβ on intracellular lysosomal pH value in primary microglia. Microglial cells were harvested and maintained in the 96-well plate, with DMEM-F12 containing 2% FBS at least for 2 h for the return of a resting state. After treated with the indicated concentration of Aβ for the indicated time, microglia cells were rinsed with live cell imaging solution. Intracellular pH of microglia was detected using fluorescent dye-based pHrodo Red AM intracellular pH indicator. The wavelength of 560 and 580 nm fluorescent light for excitation and emission was used in a Gemini EM microtiter plate reader. Forskolin (100 μM), a powerful protein kinase A (PKA) activator, capable of enhancing lysosomal acidification in intact cell, was served as control. Intracellular pH values were determined by the value of the arbitrary pH ratios of light excited at 560 nm and emitted at 580 nm with pH calibration curve kit (Invitrogen). Data were collected from 3 independent experiments with 8-paralleling wells. ^*^P < 0.05, compared with forskolin. #*P* < 0.001, compared with forskolin.

The pH value of resting microglia was around 5.96, while protein kinase A activator forskolin treatment induced a marked drop (0.71–0.86) of pH value (Figure 5). Challenge of Aβ (5 or 10 μM) induced a slight and transient drop (0.25 for 5 μM and 0.28 for 10 μM) of intracellular pH at the incubation-time of 4 h followed by sustaining pH recovery to the baseline, suggesting Aβ induced inadequate lysosomal acidification in primary microglial cells (Figure 5).

## Discussion

Abnormal protein aggregation is a universal pathogenesis of neurodegenerative diseases such as AD, Huntington's disease (HD) and Parkinson's disease (PD) (Hardy and Selkoe, 2002; Brundin et al., 2010; Zuccato et al., 2010; Jucker and Walker, 2011). Microglia is the main population in the central nervous system to phagocytize and degrade misfolded protein (Perry et al., 2010), and lysosome is the major organelle in microglia for the clearance of misfolded proteins (Nixon et al., 2008; Majumdar et al., 2011). Studies have shown that the function of lysosomal enzymes in the microglia of AD patients was potentially impaired, leading to the dysfunction in clearing misfolded Aβ and autophagy impairment (Krabbe et al., 2013), however, the underlying mechanism remained unknown (Majumdar et al., 2007). The enzyme activity in the lysosome depends on the acidification environment, impairment of which could lead to protein clearance deficiency and lysosomal storage diseases (Avrahami et al., 2013; Colacurcio and Nixon, 2016). Considering the key role in regulating lysosomal biogenesis and function, TFEB was regarded as a potential hub of misfolded protein processing (Sardiello et al., 2009; Settembre et al., 2011). Furthermore, a recent study indicated that TFEB could reduce the protein level of amyloid precursor protein (APP) and decrease the generation of Aβ (Xiao et al., 2015), suggesting a crucial role of TFEB in the generation and degredation of Aβ. In this study, we demonstrated that Aβ exerted a marked change of the cellular distribution of TFEB by enhancing its nuclear export. Moreover, OSTM1, an integral element of lysosome function, was potently inhibited by the administration of Aβ, indicating a likely cause of acidification impairment and lysosomal dysfunction. Indeed, overexpression of LAMP1 in AD brain and cerebrospinal fluid, accompanying by the dysfunction of autophagy in mice and human have been documented (Armstrong et al., 2014). Evidence from transgenic mice suggested that increased lysosomal biogenesis in activated microglia might exacerbate neuronal injury (Tanaka et al., 2013). These results supported that Aβ might lead to a decompensatory lysosomal dysfunction and resulted in the deficit of degradation by microglia in the brain.

The phenomenon of Aβ-triggered TFEB efflux out of the microglial nucleus has a vital pathological implication in the impaired degradation of misfolded protein in the microglial cells. It was reported that excessive neuronal levels of α-synuclein were closely linked to the progressive decline of lysosomal function and cytoplasmic retention of TFEB through mammalian target of rapamycin (mTOR) signaling pathway (Decressac et al., 2013). Both de-phosphorylated and phosphorylated forms of TFEB existed in nuclear compartment (Pe-a Llopis et al., 2011), and the migration of TFEB greatly influenced the function of TFEB, resulting in the facilitation of clearance of abnormal protein deposition in PD and HD (Tsunemi et al., 2012; Decressac and Björklund, 2013). Therefore, it is likely that misfolded protein in neurodegenerative diseases, like α-synuclein in PD and huntingtin in HD, might also possess the similar property in regulating TFEB expression and cellular distribution. Theoretically, the insult of exotic misfolded protein would cause a rigorous proteolysis reaction, which required the activation of lysosome biogenesis and assembly where the entrance of TFEB into nucleus was essentially needed. Unexpectedly, when microglial cells were incubated with Aβ, the distribution of TFEB appeared to be a dynamic change with the duration of incubation. There was a transient increase of TFEB in the nuclear of primary microglia, suggesting a compensatory reaction of microglia to Aβ, and then followed by a sustained efflux of TFEB from the nucleus, which was consistent to the finding in Parkinson's disease rat model (Decressac and Björklund, 2013). It is not clear now if the aberrant response of microglia toward Aβ was a unique condition or not, thus we should further test some other large molecules such as α-synuclein and huntingtin to confirm if it is a universal phenomenon.

Incomplete acidification of lysosome in microglia greatly limited the digestion of internalized Aβ (Majumdar et al., 2007; Wolfe et al., 2013). A chloride transporter CLCN7, an important protein in regulating lysosomal pH, is necessarily required for the lysosomal function and Aβ degradation in microglial lysosome (Majumdar et al., 2011). OSTM1, as the β-subunit of CLCN7, has been shown to be crucial for lysosomal trafficking of CLCN7 (Lange et al., 2006). Majumdar and his colleagues found that the expression of CLCN7 and OSTM1 in microglia surrounding senile plaque was significantly decreased in transgenic AD mice (Majumdar et al., 2011). It was reported that β-amyloid could activate microglia and induce inflammation response, indicating that cytokine TNF-α could compromise lysosome acidification (Wang et al., 2015). A recent study showed that loss of OSTM1 function resulted in severe neurodegeneration (Héraud et al., 2014). Thus, we observed the effects of Aβ on the OSTM1 expression to verify if OSTM1 play a role in the Aβ degradation. Our data showed that Aβ significantly increased the OSTM1 levels at 12 h, suggesting a compensatory activation of microglia. However, the expression of OSTM1 was dramatically suppressed by Aβ administration in primary microglial cells after incubated with Aβ for more than 24 h. It was worth to note that the status of OSTM1 level was consistent to the nucleus distribution of TFEB, and the more TFEB in nucleus, the higher OSTM1 level in cytosol. In addition, since the expression of OSTM1 and CLCN7 in osteoclasts are co-regulated by the microphthalmia transcription factor (MITF) belonging to the MiT family including MITF, TFEB, TFEC, and TFE3 (Kuiper et al., 2004; Meadows et al., 2007). Considering the similar structure of these family members, we hypothesized that OSTM1 might be the downstream molecular regulated by TFEB in microglia.

It was generally considered that the lysosomal stresses would induce TFEB nuclear recruitment and a concomitant expression of lysosomal genes (Sardiello et al., 2009; Martina et al., 2012), which was a compensatory strategy for the cell to deal with large misfolded molecules. However, we found Aβ caused a paradoxical TFEB efflux out of nuclear and the suppression of OSTM1, which may result in a declined lysosome function. The unexpected decline in the nucleus of TFEB and the marked suppression of OSTM1, coupling with inadequate lysosomal acidification, provided a putative explanation for the inability of Aβ degradation within microglia. Though we found Aβ could also significantly induced the expression of LAMP1 similar to OSTM1 at 12 h, unlike the decrease of OSTM1, the LAMP1 level continued to increase at 24 h. The aberrant increase of LAMP1 might suggest the accumulation of defective lysosome in microglia. This inconsistent result indicated that other potential transcriptional factors may be involved in LAMP1 expression. A study from Martina et al. demonstrated that TFE3 could regulate the expression of LAMP1 (Martina et al., 2014), which might be a potential compensatory pathway to increase LAMP1 expression. Further studies were critically needed to elucidate the reason of Aβ-induced excessive LAMP1 expression.

## Conclusions

The challenge of Aβ induced nuclear export of TFEB, coupled with a marked inhibition of OSTM1 expression, excessive LAMP1 production and inadequate lysosome acidification in primary microglia. Together with the finding that extracellular accumulated Aβ plaques with surrounding activated microglia develops the pathologic hallmark of AD, our study indicated that Aβ itself might lead to a disturbance of Aβ clearance in the AD brain through inhibition of lysosomal acidification, but not lysosome formation, thus forming a vicious cycle in Aβ clearance. To the best of our knowledge, since no specific agonist for TFEB nuclear translocation was developed yet, drug discovery based on this pathway would have a potential implication on the AD therapy.

## Author contributions

XG, PT, LCHE, PL, and CH performed experiments; XZ, YL, and XL designed experiments; LCHO analyzed data; XG and RL jointly drafted the manuscript. All authors read and approved this final version.

### Conflict of interest statement

The authors declare that the research was conducted in the absence of any commercial or financial relationships that could be construed as a potential conflict of interest.
