# Supplementary figures and images for: Amyloid β-Induced Redistribution of Transcriptional Factor EB and Lysosomal Dysfunction in Primary Microglial Cells
Source: Front Aging Neurosci. 2017 Jul 19;9:228. doi: 10.3389/fnagi.2017.00228 (PMC5515861; doi:10.3389/fnagi.2017.00228)

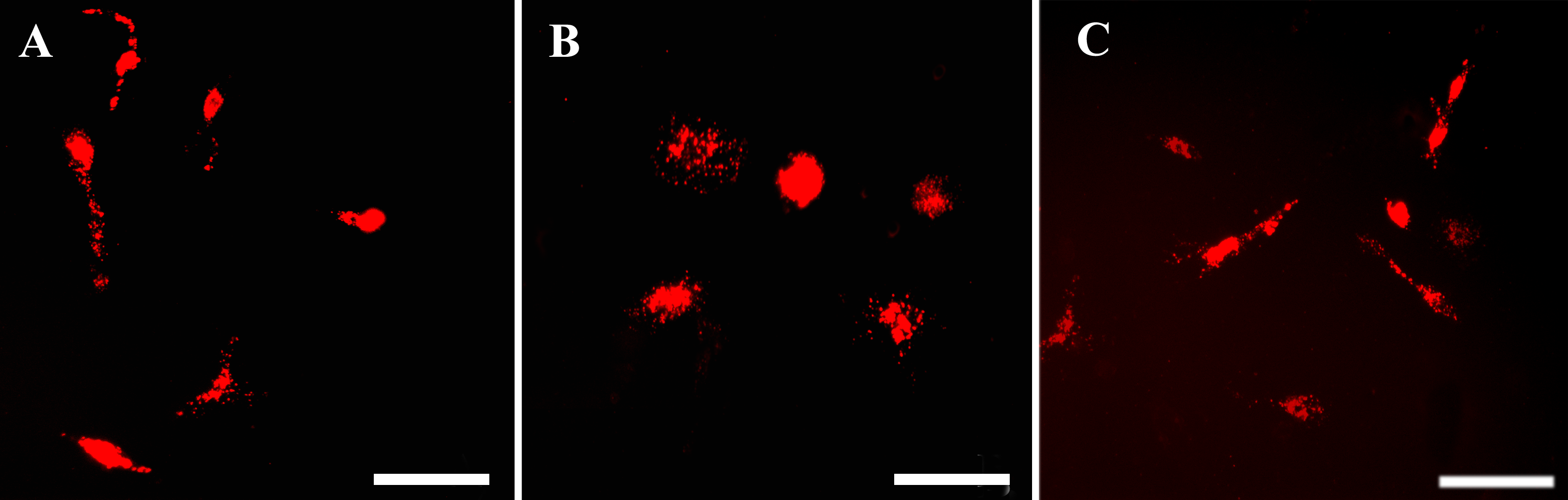

Supplement: Supplementary file 1 [file Image1.TIF]

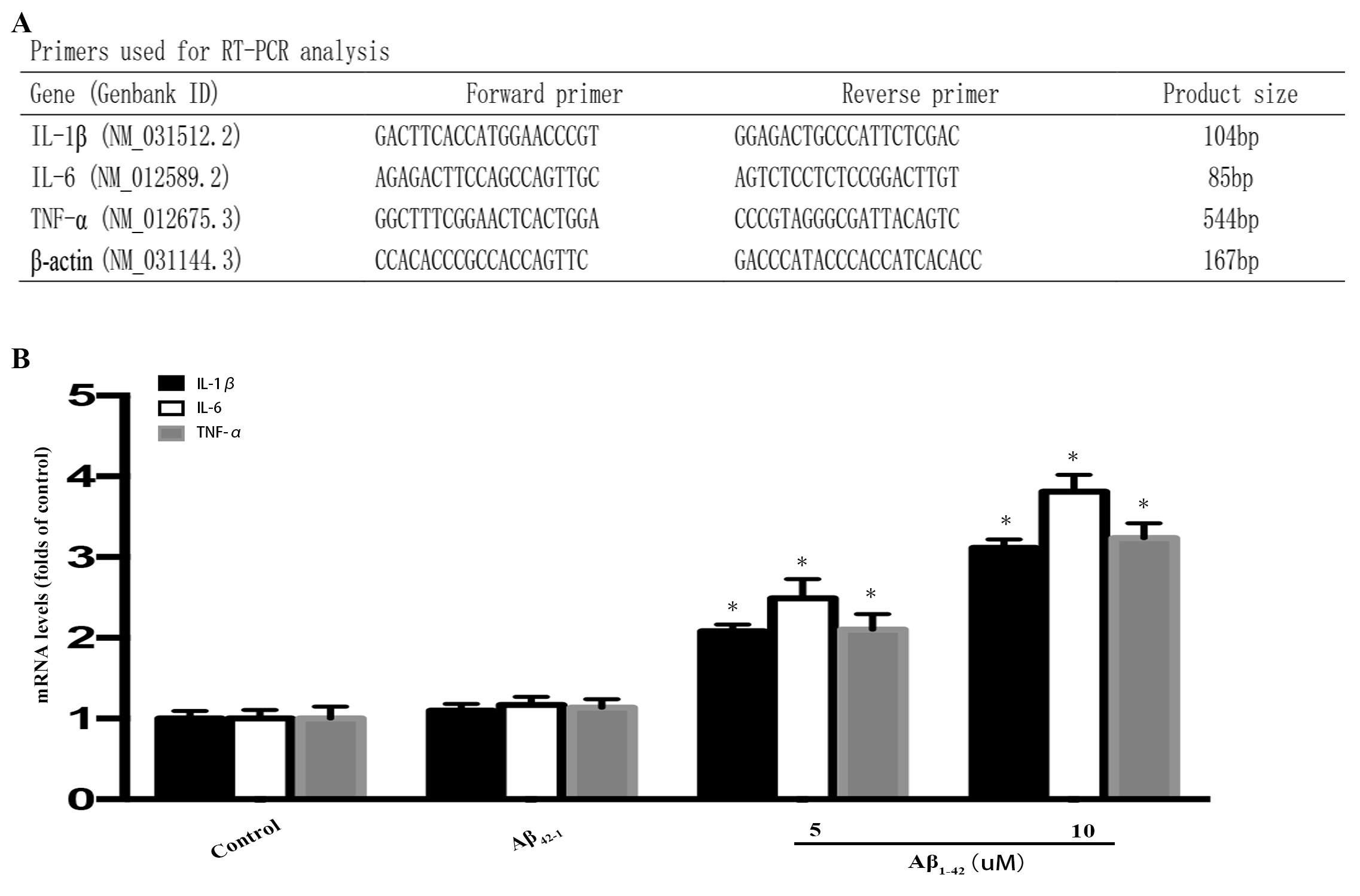

Supplement: Supplementary file 2 [file Image2.TIF]
