# Supplementary material for: Amyloid β-Induced Redistribution of Transcriptional Factor EB and Lysosomal Dysfunction in Primary Microglial Cells
Source: Front Aging Neurosci. 2017 Jul 19;9:228. doi: 10.3389/fnagi.2017.00228 (PMC5515861; doi:10.3389/fnagi.2017.00228)
Supplement: Supplementary file 3 [file Presentation1.PDF]

Fig. S1 Aβ-induced primary microglia morphological changes. Purified microglia cells were maintained in DMEM-F12 supplemented with 2% FBS for 24 h. Then $A\beta_{1-42}$ (10 μM), $A\beta_{42-1}$ (10 μM) or vehicle were added to the medium for 24 h. For morphological change, diI-Ac-LDL (5 μg/ml) was added for 4 h at 37°C and cells were visualized via fluorescence microscope. Vehicle-treated microglia appeared typically thin cell body with ramification (**A**). Most microglia developed round shape with enlarged and amoeboid cell bodies when incubated with 10 μM $A\beta_{1-42}$ (**B**) . $A\beta_{42-1}$ (10 μM) did not induce any visible morphological changes in microglia (**C**). Scale bar=50 μm.

Fig. S2 Aβ increased the mRNA levels of interleukin 1 (IL-1β), interleukin 6 (IL-6) and TNF-α in primary microglia. The primers used in the real-time PCR was presented in **A**. Purified microglia cells were maintained in DMEM-F12 supplemented with 2% FBS for 24 h. Then $A\beta_{1-42}$ (5, 10 μM), $A\beta_{42-1}$ (10 μM) or vehicle were added to the medium for 24 h. $A\beta_{1-42}$ treatment significantly increased the expression of mRNA of IL-1β, IL-6 and TNF-α in primary microglia, as compared to vehicle or $A\beta_{42-1}$ group (**B**). *P<0.05 (compared with control microglia).
